# Effect of Insecticides Imidacloprid and Alpha-Cypermethrin on the Development of Pea (*Pisum sativum* L.) Nodules

**DOI:** 10.3390/plants13233439

**Published:** 2024-12-07

**Authors:** Artemii P. Gorshkov, Pyotr G. Kusakin, Maxim G. Vorobiev, Anna V. Tsyganova, Viktor E. Tsyganov

**Affiliations:** 1 Laboratory of Molecular and Cell Biology, All-Russia Research Institute for Agricultural Microbiology, 196608 Saint Petersburg, Russia; a.gorshkov@arriam.ru (A.P.G.); pyotr.kusakin@arriam.ru (P.G.K.); vetsyganov@arriam.ru (V.E.T.); 2 Research Resource Centre “Molecular and Cell Technologies”, Saint Petersburg State University, 199034 Saint Petersburg, Russia; vorobiev.maxim@spbu.ru

**Keywords:** *Pisum sativum* L., symbiotic nodule, symbiosome, bacteroid, infection droplet, insecticides

## Abstract

Insecticides are used commonly in agricultural production to defend plants, including legumes, from insect pests. It is a known fact that insecticides can have a harmful effect on the legume–rhizobial symbiosis. In this study, the effects of systemic seed treatment insecticide Imidor Pro (imidacloprid) and foliar insecticide Faskord (alpha-cypermethrin) on the structural organization of pea (*Pisum sativum* L.) nodules and their transcriptomic activity were investigated. The plants were treated as recommended by the manufacturer (10 mg/mL for Imidor Pro and 50 µg/mL for Faskord) and twofold concentrations were used for both insecticides. Insecticides had no visible effect on the growth of pea plants. The nodules also showed no visible changes, except for the variant treated with twofold concentration of Imidor Pro. However, the dry weight of shoots and roots differed significantly in insecticide-treated plants compared to untreated plants in almost all treatments. The number of nodules decreased in variants with Imidor Pro treatment. At the ultrastructural level, both insecticides caused cell wall deformation, poly-β-hydroxybutyrate accumulation in bacteroids, expansion of the peribacteroid space in symbiosomes, and inclusions in vacuoles. Treatment with Faskord caused chromatin condensation in nucleus. Imidor Pro treatment caused hypertrophy of infection droplets by increasing the amount of matrix, as confirmed by immunofluorescence analysis of extensins. Transcriptome analysis revealed upregulation of expression of a number of extensin-like protein-coding genes in nodules after the Imidor Pro treatment. Overall, both insecticides caused some minor changes in the legume–rhizobial system when used at recommended doses, but Faskord, an enteric contact insecticide, has fewer negative effects on symbiotic nodules and legume plants; of these two insecticides, it is preferred in pea agricultural production.

## 1. Introduction

Temperate legumes are an important source of protein for food and nutritional security in many countries. They play a key role in sustainable crop production by enriching the soil with nitrogen through symbiotic nitrogen fixation. Due to this important ecological position, pests, in particular insects, represent a major constraint to legume production, with annual yield losses from arthropods estimated at about 18–26% and exceeding USD 470 billion [1]. According to FAOSTAT, insecticide use has increased by one and a half times worldwide from 2000 to 2021 due to the widespread behavior of insect pests [2]. In Russia, the use of insecticides increased sevenfold over the same period of time.

Insecticides are necessary for the effective control of insect pests [3,4,5], although they have a toxic effect on mammals [6,7], humans [8,9], and plants [10,11].

In Russia, the insecticides Faskord and Imidor Pro are used in legume production, in particular peas. Imidor Pro is a systemic insecticide for the pre-planting treatment of crop seeds against a wide range of pests, with imidacloprid as an active ingredient. Imidacloprid [1-[(6-chloro-3-pyridinyl)methyl]-N-nitro-2-imidazolidinimine] is a neonicotinoid insecticide with low persistence in soil, high insecticidal activity, and relatively low toxicity to mammals [12,13]. Neonicotinoids bind to postsynaptic nicotinic acetylcholine receptors in insects, inducing excessive stimulation of the central nervous system and leading to paralysis and death. They have a systemic action, causing the growing plant to be highly toxic to insect herbivores, which reduces or eliminates the necessity of applying aerial sprays of insecticides [14,15].

Faskord is an intestinal contact insecticide with foliar application of synthetic pyrethroid group against a wide range of pests. Alpha-cypermethrin, the active ingredient in Faskord, is the racemate of the highly active two cis-isomers [(S)-α-cyano-3-phenoxybenzyl-(1R,3R)-3-(2,2-dichlorovinyl)-2,2-dimethylcyclopropanocarboxylate and (R)-α-cyano-3-phenoxybenzyl-(1S,3S)-3-(2,2-dichlorovinyl)-2,2-dimethylcyclopropanocarboxylate] [16]. Alpha-cypermethrin, like other pyrethroid insecticides, targets both the peripheral and central nervous system of insects by affecting the voltage-gated sodium channel proteins that are located in the membranes of nerve cells [17]. By prolonging the opening of these channels, pyrethroids stimulate nerve cells to produce repetitive discharges, causing paralysis and possible death of insects [18,19].

Imidacloprid (and another neonicotinoid thiamethoxam), in a study on chickpea (*Cicer arietinum* L.) plants, significantly inhibited germination (up to 44% in 3× (300 μg kg^−1^) treatment compared with control plants), length and dry biomass (up to 56% and 67%), photosynthesis, and nodulation. Such insecticidal stress induced distortion in the root tip, intracellular oxidative damage, cell death, and adverse alteration in leaf anatomy. These two insecticides also adversely affect the nutritional quality and properties of chickpea grain [20]. The study of the toxicological effect of imidacloprid on common bean (*Phaseolus vulgaris* L.) plants showed that germination rate and seed vigor index reduced significantly only at the applied concentrations above the recommended dose. Root length, plant length, number of leaves, and number of nodules decreased in a dose-dependent manner. Chlorophyll a, b, and total chlorophyll maximum decreased by 95%, 80%, and 82%, respectively, after treating the plants with high doses of imidacloprid [21]. Furthermore, treatment with imidacloprid (140 g a.i./100 kg seeds) severely inhibited nodulation in mung bean (*Vigna radiata* (L.) R. Wilczek) plants. On day 40, the number of nodules per plant was 55.26% lower in the treated field compared to the untreated field, and on day 60 was 33.4% lower [22]. The number of multilobed nodules was increased in faba bean (*Vicia faba* L.) and pea (*Pisum sativum* L.) when treated with thiamethoxam [23,24].

Lentil (*Lens culinaris* Medik.) and watercress (*Lepidium sativum* L.) plants showed decreased weight, root, and stem development after 7-day exposure to alpha-cypermethrin, and the toxic effect of this pesticide was evident at concentrations above 100 mg/L for both species. Notably, stem growth was most sensitive to the insecticide [25]. Treatment of tomato (*Solanum lycopersicum* L.) plants with alpha-cypermethrin and imidacloprid resulted in a dose-dependent decrease in parameters such as germination, seedling vigor, and photosynthetic pigments, but at recommended doses, only alpha-cypermethrin had a stimulating effect on growth compared to the control [26].

Insecticides, including imidacloprid and alpha-cypermethrin, can also negatively affect rhizobia [27,28]. Imidacloprid (along with other pesticides) inhibited growth, disrupted morphology, and reduced membrane permeability and antioxidant-producing ability of *Mesorhizobium ciceri* [28]. However, another study on soil contamination with pesticides, including imidacloprid, showed that the composition of soybean (*Glycine max* (L.) Merr.) rhizosphere bacteria did not change [29]. Alpha-cypermethrin at low concentrations (0.1–0.5 µg/L) stimulated colony formation of *Rhizobium* sp. isolated from alfalfa (*Medicago sativa* L.), and at high concentrations (10–50 µg/L) inhibited their growth [30].

Nevertheless, there is very little data in the literature on the effect of imidacloprid and alpha-cypermethrin on legume–rhizobial symbiosis, and, to our knowledge, studies of nodule ultrastructure and transcriptome analysis have not been conducted. In this study, the effects of two insecticides on morphological and transcriptomic changes in pea symbiotic nodules were investigated. The application of Imidor Pro reduced growth and nodulation parameters and caused histological and ultrastructural changes. While Faskord application caused only minor morphological changes in nodules. The obtained data of transcriptomic analysis correlate with the revealed morphological changes.

Thus, it has been shown that the use of the foliar insecticide Faskord is preferable to the seed dressing Imidor Pro. However, further studies should clarify whether the less negative effect is related to the form of application or to the active ingredient.

## 2. Results

### 2.1. Nodulation and Plant Growth Parameters

**Figure 1:**
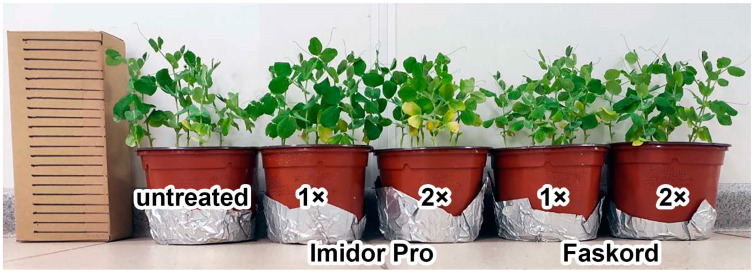
Phenotypes of pea plants (*Pisum sativum* L.) of the cv. ‘Frisson’. Untreated plants and plants treated with recommended by the manufacturer (1×), double-concentrated (2×) solutions of Imidor Pro and Faskord. Scale bars = 1 cm.

Insecticides Imidor Pro and Faskord did not affect the plant growth of the pea cv. ‘Frisson’ (Figure 1), with the exception of one variant: treatment with the twofold concentration of Imidor Pro resulted in more pronounced chlorosis of lower leaves (Figure 1).

**Figure 2:**
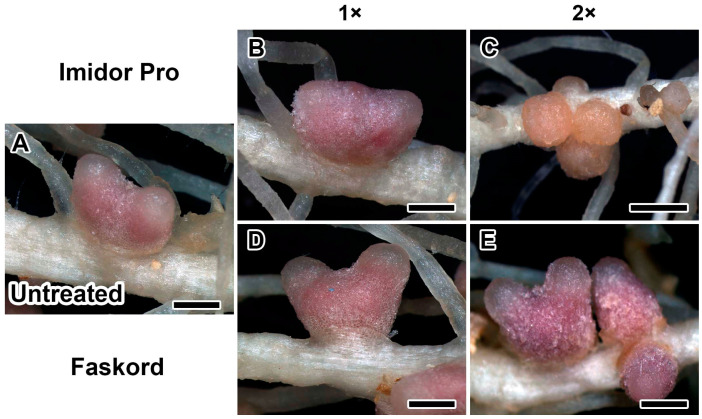
Nodule phenotypes of pea plants (*Pisum sativum* L.) of the cv. ‘Frisson’. Untreated plants (**A**) and plants treated with recommended by the manufacturer (1×; **B**,**D**) and twofold-concentrated (2×; **C**,**E**) solutions of Imidor Pro (**B**,**C**) and Faskord (**D**,**E**). Bars = 2 mm.

Nodules of plants treated with twofold concentration of insecticide Imidor Pro differed from control nodules (Figure 2). At this concentration, pea plants formed mostly brownish nodules of small size (Figure 2C). Treatment of pea plants with insecticide Faskord at both concentrations did not cause any visible changes in nodules, compared to untreated ones (Figure 2D,E).

**Figure 3:**
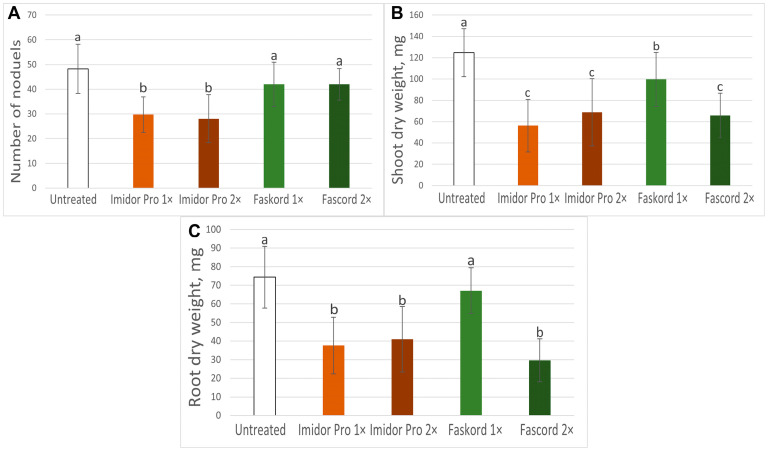
Growth parameters of pea plants (*Pisum sativum* L.) of the cv. ‘Frisson’. (**A**) Mean number of nodules per plant. (**B**) Mean shoot dry weight. (**C**) Mean root dry weight. Different letters indicate groups with a significant difference according to Tukey’s HSD test (*p*-value < 0.05; n = 20). Vertical bars represent standard deviation.

Growth and nodulation parameters were measured for plants of the cv. ‘Frisson’ treated with both insecticides. Nodule numbers followed this trend: untreated > 1× > 2×, with less nodule number in variants of treatment with Imidor Pro (Figure 3A). However, only the difference between the untreated plants and plants treated with Imidor Pro was statistically different (Figure 3A). The dry weight of plant shoots in all variants of insecticide treatment differed statistically from untreated ones. Treatment with Faskord insecticide in the recommended concentration had the least negative effect (Figure 3B). Moreover, at this concentration, the dry weight of plant roots was not significantly different from untreated ones. At the same time, in other treatments, root dry weight was decreased (Figure 3C).

### 2.2. Nodule Histological Organization

In nodules of 20-day-old untreated plants of pea cv. ‘Frisson’ (Supplementary Figure S1A) typical histological zonation of indeterminate nodules was observed. In the meristem, cells contained numerous small vacuoles, a centrally located nucleus, and basophilic cytoplasm (Supplementary Figure S1B). Numerous infection threads and droplets from which rhizobia released into the cytoplasm of plant cells were detected in the infection zone. Small juvenile bacteroids were distributed at the periphery of the cells, with the nucleus located near the central vacuole (Supplementary Figure S1C). In the nitrogen fixation zone, in mature infected cells, the nucleus lost its rounded shape and abutted to the central vacuole, and the cytoplasm was filled with numerous pleomorphic bacteroids (Supplementary Figure S1D).

**Figure 4:**
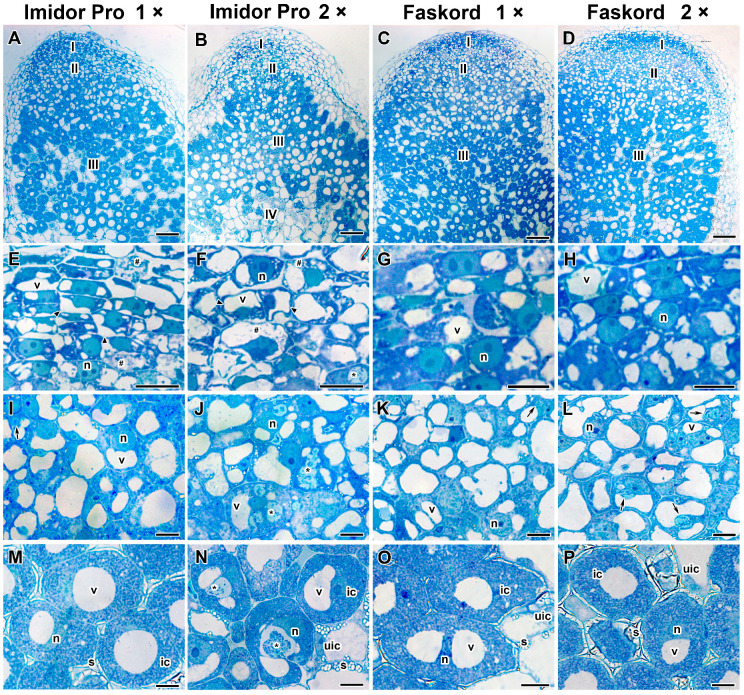
Histological organization of the nodules of pea (*Pisum sativum* L.) cv. ‘Frisson’ treated with insecticides. (**A**,**B**,**E**,**F**,**I**,**J**,**M**,**N**) Treatment with Imidor Pro. (**C**,**D**,**G**,**H**,**K**,**L**,**O**,**P**) Treatment with Faskord. (**A**,**C**,**E**,**G**,**I**,**K**,**M**,**O**) Treatment with insecticides at the concentration recommended by the manufacturer. (**B**,**D**,**F**,**H**,**J**,**L**,**N**,**P**) Treatment with a twofold-concentrated solution of insecticides. (**A**–**D**) Longitudinal section of a nodule. (**E**–**H**) Nodule meristematic cells. (**I**–**L**) Cells in the infection zone. (**M**–**P**) Infected cells in the nitrogen fixation zone. I, meristem; II, infection zone; III, nitrogen fixation zone; IV, senescence zone; n, nucleus; v, vacuole; arrows indicate infection droplets; *, enlarged infection droplets; #, cells with enlightened cytoplasm; s, starch; ic, infected cell; uic, uninfected cell. Triangles indicate vacuole fusion. Bars (**A**–**D**) = 100 µm, (**E**–**P**) = 10 µm.

Treatment of pea plants with Faskord insecticide at different concentrations and Imidor Pro insecticide at the concentration recommended by the manufacturer showed no differences from untreated plants at the histological level (Figure 4A,C,D,G–I,K–M,O,P). Although changes in meristem were observed when treated with Imidor Pro at the recommended concentration (Figure 4E). When plants were treated with a twofold concentration of Imidor Pro, in nodules, a senescence zone appeared (Figure 4B). Meristematic cells formed disordered rows; large vacuoles were formed by the fusion of small vacuoles (Figure 4E,F). Cells with enlightened cytoplasm were encountered (Figure 4E,F). The most striking feature of infected cells in the infection and nitrogen fixation zones was an increase in the size of infection droplets (Figure 4J,N). In some cells, the infection droplet could occupy most of the cell volume (Figure 4J). It should also be noted that starch accumulation was not observed in infected cells in the nitrogen fixation zone at a twofold concentration of Imidor Pro (Figure 4N) in contrast to the other treatments (Figure 4M,O,P).

### 2.3. Ultrastructure of Nodules

**Figure 5:**
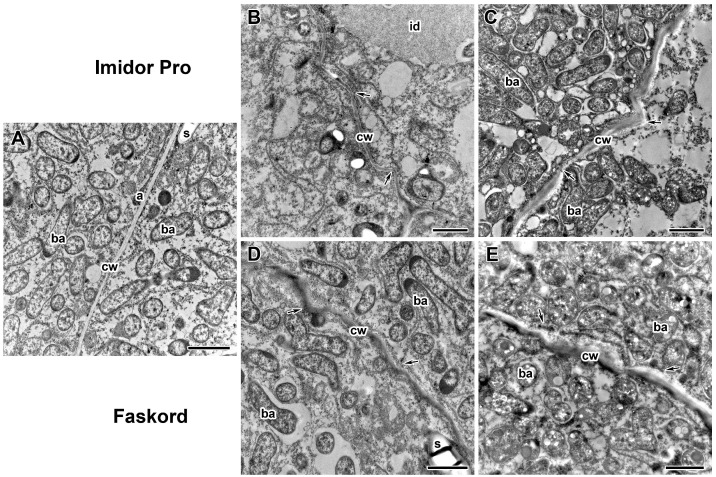
Ultrastructural organization of cell walls in nodules of pea (*Pisum sativum* L.) cv. ‘Frisson’. (**A**) Section of a nodule of an untreated 20-day-old plant. (**B**,**C**) Treatment with insecticide Imidor Pro. (**D**,**E**) Treatment with insecticide Faskord. Treatment with concentration recommended by the manufacturer (**B**,**D**) and with a twofold-concentrated solution of insecticides (**C**,**E**). cw, cell wall; ba, bacteroid; s; starch; a, amyloplast; id, infection droplet. Arrows indicate different cell wall abnormalities (curvature, swelling, uneven density). Bars (**A**–**E**) = 2 µm.

**Figure 6:**
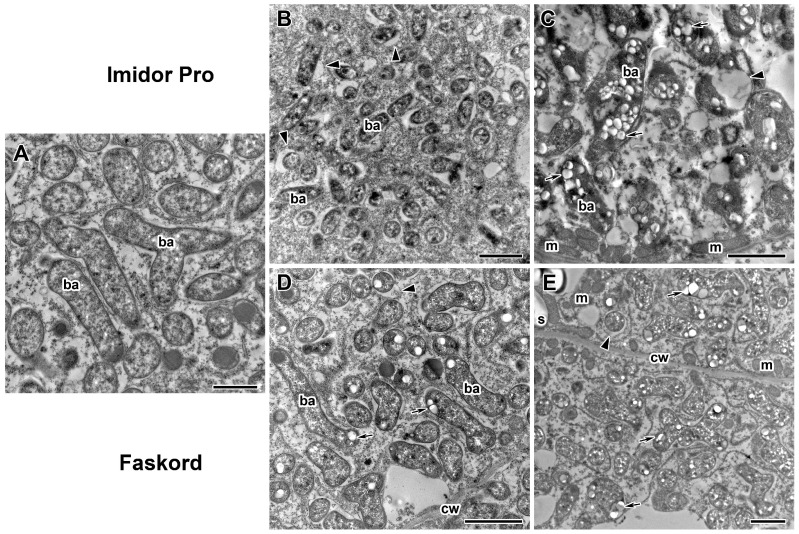
Ultrastructural organization of bacteroids in infected cells in nodules of pea (*Pisum sativum* L.) cv. ‘Frisson’. (**A**) Section of a nodule of an untreated 20-day-old plant. (**B**,**C**) Treatment with insecticide Imidor Pro. (**D**,**E**) Treatment with insecticide Faskord. Treatment with concentration recommended by the manufacturer (**B**,**D**) and with a twofold-concentrated solution of insecticides (**C**,**E**). cw, cell wall; m, mitochondrion; ba, bacteroid; s, starch. Arrows indicate poly-β-hydroxybutyrate drops in bacteroids; arrowheads indicate the expansion of the peribacteroid space in symbiosomes. Bars (**B**–**E**) = 2 µm, (**A**) = 1 µm.

**Figure 7:**
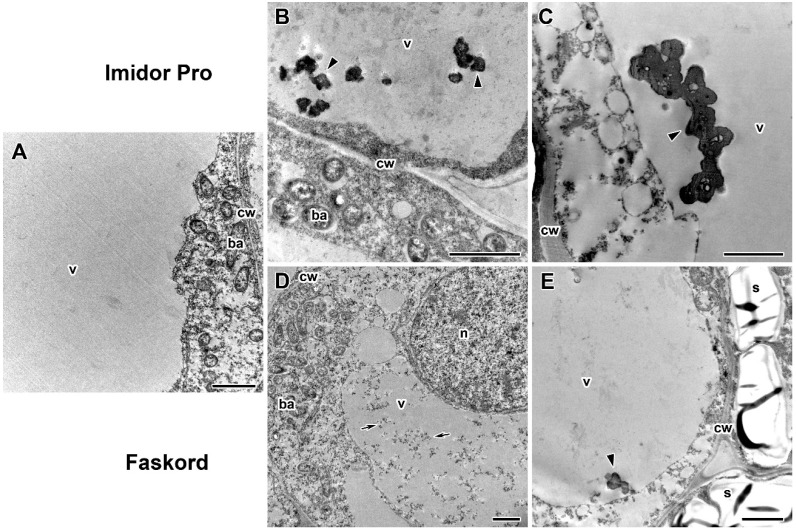
Ultrastructural organization of vacuoles in nodules of pea (*Pisum sativum* L.) cv. ‘Frisson’. (**A**) Nodule section of an untreated 20-day-old plant. (**B**,**C**) Treatment with insecticide Imidor Pro. (**D**,**E**) Treatment with insecticide Faskord. Treatment with concentration recommended by the manufacturer (**B**,**D**) and with a twofold-concentrated solution of insecticides (**C**,**E**). n, nucleus; cw, cell wall; ba, bacteroid; v, vacuole; s, starch. Arrows indicate protein complexes in vacuoles; triangles indicate inclusions in uninfected cells, presumably of a phenolic nature. Bars (**A**–**E**) = 2 nm.

**Figure 8:**
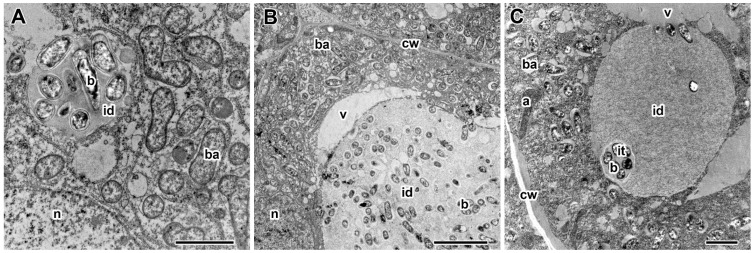
Ultrastructural organization of infection droplets in infected cells in nodules of pea (*Pisum sativum* L.) cv. ‘Frisson’. (**A**) Nodule section of an untreated 20-day-old plant. (**B**,**C**) Treatment with insecticide Imidor Pro with concentration recommended by the manufacturer (**B**) and with a twofold-concentrated solution of insecticides (**C**). n, nucleus; it, infection thread; id, infection droplet; v, vacuole; cw, cell wall; ba, bacteroid; b, bacterium; a, amyloplast. Bars (**B**) = 5 µm, (**A**,**C**) = 2 µm.

**Figure 9:**
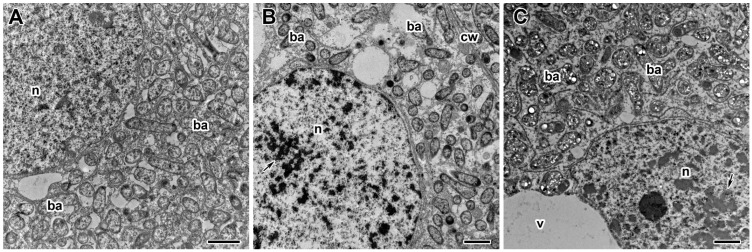
Ultrastructural organization of cell nuclei in nodules of pea (*Pisum sativum* L.) cv. ‘Frisson’. (**A**) Nodule section of an untreated 20-day-old plant. (**B**,**C**) Treatment with insecticide Faskord with concentration recommended by the manufacturer (**B**) and with a twofold-concentrated solution of insecticides (**C**). n, nucleus; cw, cell wall; ba, bacteroid; v, vacuole. Arrows indicate the formation of coarse chromatin clumps in the nuclei. Bars (**A**–**C**) = 2 µm.

To further characterize the morphological changes, ultrastructural analysis of nodules of pea plants cv. ‘Frisson’ treated with both insecticides was performed (Figure 5, Figure 6, Figure 7, Figure 8 and Figure 9). The nodules of 20-day-old control plants had an ultrastructural organization characteristic of indeterminate nodules [31,32]. In the infection zone, infected cells contained a central vacuole (Figure 7A), a large nucleus, infection threads or droplets (Figure 8A), and small juvenile bacteroids located at the cell periphery (Figure 7A). In the nitrogen fixation zone, infected cells were filled with numerous symbiosomes containing a single bacteroid (Figure 5A, Figure 6A and Figure 9A).

Treatment with Imidor Pro and Faskord insecticides resulted in both common and specific ultrastructure abnormalities. One of the common ultrastructural abnormalities was a change in cell wall structure (Figure 5B–E). When treated with both insecticides at concentrations recommended by the manufacturer, the cell walls were curved (Figure 5B,D), which was also observed at higher concentrations (Figure 5C,E). With increasing insecticide concentrations, the cell walls swelled with the appearance of areas of irregular density (Figure 5C,E). Application of Faskord insecticide at both concentrations resulted in a more electron-dense cell wall compared to the control and Imidor Pro treatments (Figure 5D,E). It should also be noted that in all variants with insecticide treatments, thinning of the cell wall at the sites of its curvature was observed (Figure 5B–E).

Transmission electron microscopy showed that bacteroids in symbiosomes of infected pea nodule cells underwent some morphological changes when treated with insecticides (Figure 6B–E) compared to control plants (Figure 6A). Poly-β-hydroxybutyrate (PHB) accumulation was observed in bacteroids after the application of Faskord at both concentrations, and more intense accumulation was observed at the twofold concentration (Figure 6D,E). Imidor Pro insecticide treatment resulted in the appearance of PHB droplets only at double concentration (Figure 6C). However, it was more intense compared to treatment with Faskord at the same concentration. Treatment with both insecticides at both concentrations led to the expansion of the peribacteroid space in symbiosomes of some cells (Figure 6B–E), an increase in the concentration of insecticides caused it to be more pronounced. This abnormality was mainly observed in juvenile bacteroids in the infection zone (Figure 6B).

Another common abnormality for variants treated with different insecticides was the accumulation of different substances in the vacuoles of nodule cells (Figure 7B–E) in comparison to untreated plants (Figure 7A). Putative protein complexes were observed in the vacuoles of individual infected cells (Figure 7D). A few vacuoles in a very small number of uninfected cells, mainly in the nitrogen fixation zone, contained inclusions of unclear composition, presumably of a phenolic nature, in response to the use of insecticides (Figure 7B,C,E).

A specific abnormality for Imidor Pro insecticide was also found (Figure 8). Hypertrophied infection droplets with increased matrix volume were found in nodules of insecticide-treated plants (Figure 8B,C). At the same time, extremely low numbers of bacteria were observed in some infection droplets, resulting in the enlargement of infection droplets only due to matrix (Figure 8C). It is worth noting that the matrix of infection droplets became more granular after insecticide treatment (Figure 8B,C). Higher concentrations of insecticide resulted in more of these altered infection droplets.

A Faskord-specific morphological feature was found (Figure 9B,C): coarse chromatin clumps appeared in the nuclei of some infected cells (Figure 9B) of nodules already at the recommended by the manufacturer concentration. With increasing concentration, such cells were more frequent.

Nevertheless, other organelles (mitochondria (Figure 6C,E) and plastids (Figure 8C)) were not affected by the studied insecticides.

### 2.4. Immunocytochemical Analyses

**Figure 10:**
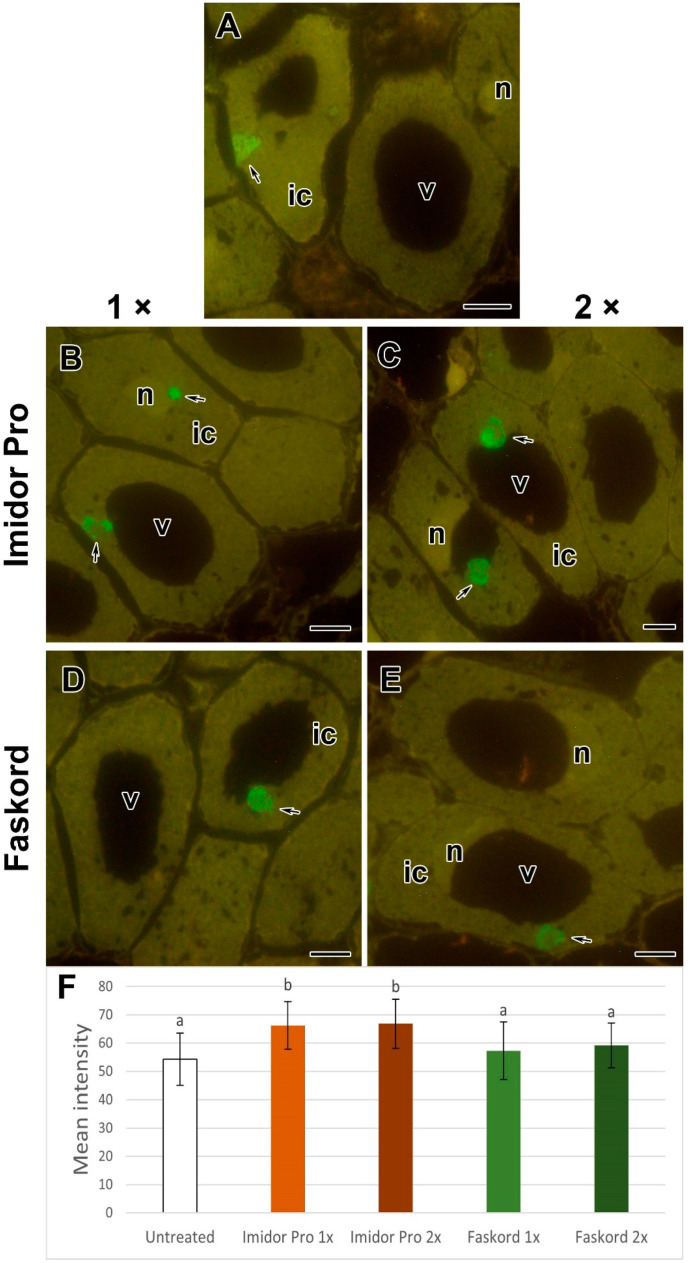
Effect of insecticide treatment of pea (*Pisum sativum* L.) cv. ‘Frisson’ on the matrix composition of infection droplets in nodule cells. (**A**–**E**) Immunolocalization of extensin labeled with JIM11 monoclonal antibody (MAb). (**A**) Section of nodules of untreated plants. Treatment with Imidor Pro (**B**,**C**) and Faskord (**D**,**E**) with concentration recommended by the manufacturer (**B**,**D**) and with a twofold-concentrated solution of insecticides (**C**,**E**). (**F**) Mean fluorescence intensity. The secondary antibodies used were goat anti-rat (**A**–**E**) IgG MAb conjugated with Alexa Fluor 488. ic, infected cell; n, nucleus; v, vacuole. Arrows indicate infection droplets. Different letters indicate groups with a significant difference according to Tukey’s HSD test (*p*-value < 0.05; n = 30–45). Vertical bars represent standard deviation. Bars = 10 µm.

For a more detailed study of the matrix composition of infection droplets in nodule cells, the immunocytochemical analyses were performed using monoclonal antibodies (MAbs) to glycoprotein epitopes of extensin JIM11 (Figure 10A–E) and arabinogalactan protein-extensin MAC265 (Supplementary Figure S2A–E). It was shown that in nodules of treated plants, the intensity of fluorescence associated with glycoprotein labeled with JIM11 statistically significantly increased in variants treated with Imidor Pro insecticide (Figure 10F) compared to untreated plants (Figure 10F). Nevertheless, at the same time, in variants treated with Faskord insecticide, the intensity of JIM11 label (Figure 10D–F) stayed level with untreated plants. However, the intensity of the epitope labeled with MAC265 was not significantly increased (Supplementary Figure S2B–F), compared to the untreated variant (Supplementary Figure S2A,F).

### 2.5. Transcriptome Analysis

Treatment of Frisson plants with the insecticide Imidor Pro at the concentration recommended by the manufacturer affected 133 genes: 98 were upregulated, and 35 were downregulated (Supplementary Table S1). Among upregulated genes notable was an abundance of cell-wall-related genes coding leucine-rich repeat extensin-like protein (Psat7g168680); fasciclin-like arabinogalactan proteins (Psat1g194520, Psat7g068160, Psat7g224400, Psat7g068120, Psat5g277720, Psat5g277760); involved in lignin metabolism laccase 4-like (Psat7g247800), coniferyl alcohol acyltransferase (Psat2g144000), and dirigent protein (Psat3g124040); COBRA-like proteins (Psat3g204320, Psat1g128520); glucan 1,3-beta-glucosidase A (Psat2g074320); catalytic subunits 4 (Psat1g149960), 7 (Psat6g061600, Psat4g125440, Psat7g006400), and 8 (Psat7g068960) of cellulose synthase; galacturonosyltransferase (Psat5g249800). Other upregulated genes included coding detoxification proteins (Psat5g092480, Psat2g101000); hormones-related cytochromes P450 (Psat5g016280 and Psat6g021360), SAM-dependent carboxyl methyltransferase (Psat4g015520), cytokinin dehydrogenase (Psat6g021360), oxidoreductase (Psat1g152520); CDR1-like aspartic proteinase (Psat2g026280). Among downregulated genes were those such as amino acid transporter (Psat5g133080, homolog of MtUMAMIT22), glycosyl hydrolase family 18 acidic endochitinase (Psat1g131280), negative regulators of jasmonate response (Psat1g194920, Psat0s766g0040), and phenylalanine ammonia-lyases (PALs: Psat3g023040, Psat3g023120, Psat1g046920).

## 3. Discussion

### 3.1. Effects of Insecticides on Plant Growth and Nodulation

In this study, the effects of two insecticides (with imidacloprid or alpha-cypermethrin as active ingredients) on pea plant growth and nodulation were investigated. Only Imidor Pro at a twofold concentration caused visible changes in plants (Figure 1). However, the dry weight of shoots and roots of pea plants decreased in all treatments, except for the Faskord treatment at the recommended concentration (Figure 3B,C). Previously, a triple dose of imidacloprid (300 mg kg^−1^) reduced seed germination by 44% in vitro and by 20% in pots, total length, dry weight of shoots and roots of chickpea by 56%, 48%, and 52%, respectively [20]. The toxic effect of alpha-cypermethrin on lentil and watercress plant growth and weight (stem and root) was also evident at concentrations above 100 mg/L [25]. In peas, insecticides fipronil and pyriproxyfen decreased the dry weight of plants, as well as seed yield, especially at a threefold concentration (0.6 and 3.9 mg kg^−1^ soil, respectively) [33]. At the same time, the insecticide Gaucho 600 FS (with the active ingredient imidacloprid) had no suppressive effect on soybean root length when seeds were pretreated at concentrations of 1, 2 and 3 L/100 kg seed [34].

Insecticides Imidor Pro at the concentration recommended by the manufacturer and Faskord in all concentrations did not cause any visible alterations in nodules (Figure 2B,D,E). However, a double dose of Imidor Pro caused small brownish, likely ineffective, nodules to appear (Figure 2C). Moreover, the application of imidacloprid (Imidor Pro) already at the recommended concentration reduced the number of nodules (Figure 3A). Imidacloprid, acetamiprid and thiamethoxam (neonicotinoids insecticides) treatments of faba bean plants after 15 days from planting resulted in the reduction of the nodule number up to 31.2, 18.7, and 12.5%, respectively. Additionally, imidacloprid treatment reduced the dry weight of nodules by up to 7% [35]. Imidacloprid also strongly suppressed nodulation in mung bean in field trials [22]. The number of nodules, nodule dry biomass, and leghemoglobin content in chickpea plants treated with imidacloprid consistently decreased with subsequent increases in insecticide concentration. The triple concentration had a stronger toxic effect on nodule number and nodule dry biomass, maximally reducing them by 76% and 61% [20]. Fipronil at the recommended concentration reduced nodule number and dry weight by 5 and 17%, respectively, in lentil plants, and at threefold concentration reduced both parameters by 37% as compared to the control [36]. When different legumes were studied, fipronil reduced the number of nodules (percent reduction compared to control) in each legume plant in the following order: pea (44) > chickpea (19) > mung bean (10) > lentil (5) [37]. Treatment with alpha-cypermethrin (Faskord) did not affect the number of nodules on pea plants (Figure 3A). To our knowledge, this is the first report on the effect of alpha-cypermethrin on nodulation. It is interesting to note that treatment of barrel medic (*Medicago truncatula* Gaertn.) plants with another pyrethroid insecticide based on deltamethrin (15 mg/m^2^) resulted in a reduction of nodule biomass [38].

### 3.2. Alterations on Histological and Ultrastructural Levels

There are no histological or ultrastructural data in the literature, to our knowledge, on the effects of insecticides on legume nodules. In this study, prominent histological changes in pea nodules were observed only when treated with Imidor Pro insecticide at double concentration: disordered rows of meristematic cells and overgrowth of infection droplets in the infection and nitrogen fixation zone (Figure 4B,F,J,N), as well as the formation of an early senescence zone (Figure 4B). Previous studies have shown significant changes in the histological organization of pea nodules when exposed to fungicide tetramethylthiuram disulfide (TMTD) [32], triazole fungicides Titul Duo and Vintage [39], and to glyphosate-based herbicide Sprut Extra [40]. It was shown that various abiotic stress factors (such as elevated temperature, heavy metals, nitrates, drought, and salinity) lead to the formation of a senescence zone [41,42,43,44,45].

Treatment with Imidor Pro and Faskord insecticides resulted in both common and specific ultrastructure abnormalities in pea nodules. The most striking common ultrastructure abnormality was cell wall structure alteration (Figure 5B–E). Already at the recommended insecticide concentrations, curving and thinning of cell walls occurred (Figure 5B,D), which were observed at double concentrations as well (Figure 5C,E). Elevated insecticide concentrations also induced the appearance of swollen cell walls with uneven density (Figure 5C,E). It should be noted that the application of Faskord insecticide at both concentrations resulted in a more electron-dense cell wall compared to untreated pea plants and Imidor Pro treatment (Figure 5D,E). Previously, it was shown that cell wall ultrastructure changes in response to different external factors, including pesticides. Indeed, swelling, thinning, and distortion of cell walls in pea nodules were observed after treatment with different fungicides and herbicides [32,39,40]. Treatment with Simazine herbicide caused the appearance of malformed infected cells whose cell walls had high electron density [46]. Excessive exposure of lupin (*Lupinus albus* L.) nodules to copper caused cell wall deformation in the cells of the outer cortex [47]. Exposure of pea plants to NaCl led to the formation of wrinkled cells with disorganized cell walls in nodules [48]. Cell wall swelling was observed in nodules of mutant line SGEFix^−^-7 (*sym27*) under short-term (3 days) exposure to elevated temperature [49].

The insecticide treatment of pea plants affected not only the cell walls but also the bacteroids and symbiosomes (Figure 6B–E), compared to untreated plants (Figure 6A). PHB granules were observed in bacteroids after treatment with both insecticides at double concentration (Figure 6C,E) and when treated with Faskord at the recommended concentration (Figure 6D). The use of Imidor Pro and Faskord resulted in the peribacteroid space expansion at both insecticide concentrations (Figure 6B–E). Previously, the treatment of pea plants with pesticides [32,39,40] and exposure of black medick (*Medicago lupulina* L.) plants to excessive Cu [41] led to the enlargement of peribacteroid space in symbiosomes. In addition, symbiosomes in pea nodules with enlarged peribacteroid space were observed after 24 h of exposure to elevated temperature [42,49]. Aluminum stress in barrel medic caused spherical membrane bulges to appear in young symbiosomes just released from the infection thread [50]. PHB accumulation in bacteroids is activated by various stressors in nodules of different legumes [50,51,52]. In particular, in pea nodules, PHB accumulated in bacteroids in response to cadmium [53,54], fungicides [32,39] and elevated temperature [42,49].

Treatment of pea plants with Imidor Pro and Faskord caused vacuole-associated abnormalities (Figure 7B–E). Protein complexes were likely observed in the vacuoles of individual infected cells in response to Faskord treatment (Figure 7D). Inclusions, presumably of phenolic composition, accumulated in response to insecticide use at both concentrations (Figure 7B,C,E). Similar phenolic inclusions in pea nodules were observed after treatment with fungicides [32,39], heat [42] and saline stresses [55].

The enlargement of infection droplets was a specific feature for nodules of Imidor Pro-treated plants (Figure 8B,C). In some infected cells, the infection droplet occupied almost the entire cell (Figure 8B). This study is probably the first to report the negative effect of pesticides on infection droplets in legume nodules. However, a similar increase in infection droplets was described in symbiotic mutants. Indeed, hypertrophic infection droplets were observed for pea and barrel medic mutants in orthologous genes *Sym40* and *EFD* [31,56,57], in 28-day-old white nodules of the pea mutant SGEFix^−^-2 (*sym33–3*) [58], and *nip* mutant of barrel medic [59].

A specific abnormality in the form of chromatin condensation was observed in nodules of pea plants treated with Faskord insecticide at both concentrations (Figure 9B,C). Previously, chromatin condensation was demonstrated in pea nodules exposed to elevated temperature [42] and treated with herbicides Sprut Extra and Forward [40]. RNAi-mediated silencing of *GmFWL1* expression in soybean plants resulted in decreased nuclear size and changes in chromatin structure [60]. The *npd1* mutant of barrel medic, characterized by arrest of nodule development at an intermediate stage, also displayed chromatin condensation [61].

To study the composition of the matrix of infection droplets in nodule cells of pea plants, immunocytochemical analysis was performed. It showed that the fluorescence intensity associated with JIM11 labeled extensin was significantly increased in the nodules of Imidor Pro-treated plants (Figure 10B,C,F) compared to untreated plants (Figure 10A). However, at the same time, in variants treated with Faskord insecticide, the intensity of JIM11 label was not changed (Figure 10D–F). This may suggest that extensin labeled with JIM11 are involved in the response to Imidor Pro. Fluorescent immunolocalization of nodules of mutants SGEFix^−^-1 (*sym40–1*) and *efd*–*1* also showed the presence of a JIM11-recognisable epitope in infection droplets, although intensity quantification in that study was not performed [62]. It is worth noting that the intensity of the epitope labeled with MAC265 was not significantly increased (Supplementary Figure S2). This MAb is used to label the matrix of the infection thread [63]. The lack of difference in fluorescence intensity between nodule cells of untreated and insecticide-treated pea plants may indicate that the matrix of infection threads has not changed.

### 3.3. Transcriptomic Changes in Imidaclorpid-Treated Plants

Studies of insecticide effects on plants at the transcriptomic level are rare. Indeed, soybean trifoliate after 10 days post-treatment with imidacloprid showed a decrease in genes involved in cell wall biosynthesis, phenylpropanoid, and phytohormones pathways [64], while pepper (*Capsicum annuum* L.) plants, after 30 days of growth in treated soil, showed suppression of secondary metabolism, including flavones, phenolic acids, and phytohormones [65]. In this study, imidacloprid caused ultrastructural abnormalities in nodules similar to those caused by treatments with fungicides and herbicides [32,39,40]. However, studied pesticides led to different responses at the transcriptomic level (Supplementary Table S1). Interestingly, treatment with imidacloprid resulted in enlarged infection droplets, while several genes for arabinogalactan proteins containing the fasciclin domain were upregulated. Currently, the function that arabinogalactan proteins of this group may play in legume–rhizobial symbiosis is not entirely clear, but their involvement in crosslinking and cell wall adhesion in plants has been suggested [66] as well as in swelling, interpolymer connectivity, secondary cell wall formation [67]. Interestingly, for other arabinogalactan proteins, arabinogalactan protein-extensins, a role in the formation of the matrix of infection threads and droplets has been shown [68]. Probably, the increased gene expression of cellulose synthase subunits and COBRA-like proteins involved in the orientation of microfibrils is related to the observed cell wall abnormalities. The effect of treatment on lignin metabolism was observed: genes related to its degradation (laccase 4, Psat7g247800) and synthesis (coniferyl alcohol acyltransferase, Psat2g144000) were upregulated, while several PAL genes were downregulated. A decrease in lignin biosynthesis is associated with an increase in nodulation in alfalfa [69], whereas an increase in its content during nodulation is associated with plant defense responses to rhizobial colonization [70]. PALs are not only involved in lignin biosynthesis but also control an important step in secondary metabolism [71]. Elevated levels of many PAL genes, including homologs of genes from this study, were observed in nodules of *Lotus japonicus* (Regel) K. Larsen [72] and alfalfa [70]. It is likely that the increased expression of the leucine-rich repeat extensin-like protein 4 gene is associated with the observed morphological and transcriptional changes in the cell wall. Similar proteins of this family, characterized by the presence of both extensin and kinase domains, are hypothesized to be able to directly sense cell wall alterations, as shown for the salt-stressed *Arabidopsis thaliana* (L.) Heynh. [73].

In this study, imidacloprid treatment caused an increase in the expression of genes probably involved in strigolactone synthesis: cytochrome Psat5g016280 [74]; SAM-methyltransferase Psat2g133560, a homolog from the SABATH family of which in *A. thaliana* AT4G36470 is involved in the last step in the synthesis of the biologically active strigolactone-like substance methyl carlactonoate [75]; Psat6g021360, which homolog in *A. thaliana* [76] and soybean *MAX1* [77] is involved in the final step of the synthesis of canonical strigolactones; and Psat1g152520, whose homolog in *A. thaliana* AT3G21420 is involved in the synthesis of non-canonical strigolactones [78]. The role of strigolactones in nodulation is not completely clear, but their negative effect on the function of the mature pea nodule has been shown [79]. A negative effect on the nodule is also known for jasmonic acid [80]. Interestingly, the jasmonic acid response repressor genes of the TIFY family, for which increased expression has been shown in peanut (*Arachis hypogaea* L.) nodules [81], decreased their expression in response to imidacloprid treatment. Together, this may indicate suppression of nodule function by the insecticide treatment.

## 4. Materials and Methods

### 4.1. Plant Material and Bacterial Strain

The pea (*Pisum sativum* L.) commercial mid-ripening cv. ‘Frisson’ [82] was used. This cultivar was characterized by determinate flowering habit, white flowers, and it is cultivated in many European countries. The streptomycin-resistant *Rhizobium johnstonii* strain 3841 [83] (former *R. leguminosarum bv. viciae* [84]) was used for inoculation. Bacteria were grown on a solid TY medium [85] at 28 °C with the addition of streptomycin at a concentration of 600 µg/mL.

### 4.2. Inoculation and Plant Growth Conditions

Pea seeds were sterilized with concentrated sulfuric acid for 30 min and washed with sterile water 10 times. The seeds were planted in pots with vermiculite immediately after sterilization, and then each seed was inoculated with 1 mL of an aqueous suspension of bacteria (10^7^–10^8^ cells). Plants were grown in vermiculite moistened with a nitrogen-free nutrient solution [86] in a growth chamber (MLR-352H, Sanyo Electric Co., Ltd., Moriguchi, Japan) under controlled conditions: day/night, 16/8; temperature 21 °C; humidity 75%; illumination 280 mol photons m^−2^ s^−1^, watering with filtrated water every 3 days to ensure optimal substrate moisture (70% *w/w*). The commercial solution of the insecticide Imidor Pro contained 200 g/L of imidacloprid; Faskord contained 100 g/L of alpha-cypermethrin. Treatment of plants with insecticides was carried out in concentrations recommended by the manufacturer: 10 mg/mL for Imidor Pro and 50 µg/mL for Faskord, as well as twofold concentrations. Spraying of plant leaves with Faskord was carried out on the 10 days after inoculation (DAI) with a manual sprayer and plants were harvested on the 10th day after treatment. Seed treatment with Imidor Pro was carried out by shaking in Petri dishes with the insecticide working solution and plants were harvested on the 20th DAI. Thus, 20-day-old plants were analyzed. 

### 4.3. Phenotypic Analysis of Plants and Nodules

Growth and nodulation parameters were analyzed for 20 plants for each variant. Nodules were counted immediately after washing the plants from vermiculite. For weight measurements, cotyledons were removed, shoots and roots were separated, and then dried in a Memmert UF160 oven (Memmert GmbH, Schwabach, Germany) at 40 °C. Pea nodules were photographed using a SteREO Lumar.V12 stereo microscope equipped with an AxioCam MRc 5 camera (Carl Zeiss, Oberkochen, Germany).

### 4.4. Statistical Analysis

Statistical data analysis was carried out using the software STATISTICA version 10 (StatSoft, www.statsoft.com). For phenotypic analysis of plants and nodules, and mean fluorescence intensity, statistically significant differences were assessed using one-way ANOVA (*p* < 0.05) and Tukey’s HSD test (*p* < 0.05).

### 4.5. Microscopy

For analyses, 15 nodules from 10 plants were harvested. Sample preparation, sectioning, contrasting for electron microscopy, and staining for light microscopy were performed as previously described [39]. Semi-thin sections were analyzed using an Axio Imager.Z1 microscope (Carl Zeiss). Photographs were taken with an Axiocam 506 digital camera (Carl Zeiss). Ultrathin sections were viewed with a JEM-1400 EM transmission electron microscope (JEOL Ltd., Tokyo, Japan) equipped with a Veleta CCD camera (Olympus, Münster, Germany).

For immunofluorescence microscopy, semi-thin sections were prepared as described by Gorshkov et al. [39]. Sections were analyzed using an Axio Imager.Z1 microscope (Carl Zeiss) equipped with an Axiocam 506 digital camera (Carl Zeiss), X-Cite 120Q UV light source (Excelitas Technologies, Ontario, Canada), and a Zeiss filter set 09 (λ_ex_ = 450–490, λ_em_ = 515 nm). The following MAbs were used as primary antibodies: JIM11, recognizing hydroxyproline-rich glycoprotein with four arabinose residues (Hyp-Araf_4_), such as extensins [87,88,89]; and MAC265, recognizing chimeric arabinogalactan-protein extensin [63,90]. Goat anti-rat IgG MAb conjugated with Alexa Fluor 488 (Agrisera, Vännäs, Sweden) were used as secondary antibodies.

Morphometrical data were obtained as described previously [39]. During the processing of fluorescent images, the areas of the presence of a signal and its absence were selected, the average fluorescence intensity was identified using ImageJ built-in functionality, and the average fluorescence intensity of the signal was normalized to the average intensity of the area without a signal.

### 4.6. RNA Sequencing and Transcriptome Analysis

RNA extraction from nodules of 20-day-old cv. ‘Frisson’ plants treated with Imidor in the recommended concentration was conducted as previously described [39]. CeGaT GmbH (Tübingen, Germany) performed assessment of RNA quality, library preparation using a TruSeq Stranded Total RNA with Ribo-Zero kit (Illumina, San Diego, CA, USA), paired-end sequencing with a NovaSeq 6000 (Illumina), demultiplexing by Illumina bcl2fastq version 2.20 and adapters trimming with Skewer version 0.2.2 [91]. Quality assessment of the resulting reads, filtering, and mapping to the reference genome [92], and subsequent bioinformatics analyses were carried out as previously described [93].

## 5. Conclusions

Testing and selecting pesticides that do not impair symbiotic nitrogen fixation is one of the most preferable ways to improve agricultural production. In the present study, it was shown that both tested insecticides resulted in cell wall modification, PHB accumulation in bacteroids, expansion of the peribacteroid space in symbiosomes and the appearance of various inclusions in vacuoles, and chromatin condensation when treated with Faskord insecticide, which has already been shown for other pesticides. At the same time, Imidor Pro-specific abnormalities associated with changes in the morphology of infection droplets were observed. In general, seed treatment with Imidor Pro insecticide is not desirable as it negatively affects both plant parameters and nodulation. Of the two insecticides studied, the insecticide Faskord appears to be preferable for use in pea crop production, as its application does not cause visible changes in the growth of plants and symbiotic nodules.

## Data Availability

The original contributions presented in this study are included in the article/Supplementary Material. Further inquiries can be directed to the corresponding author. The transcriptomic data presented in this study are openly available at NCBI SRA under the accession number PRJNA1193277.

## Supplementary Materials

The following supporting information can be downloaded at: https://www.mdpi.com/article/10.3390/plants13233439/s1, Figure S1: Histological organization of the nodules of 20-day-old untreated plants of pea (*Pisum sativum* L.) cv. ‘Frisson’; Figure S2: Effect of insecticide treatment of pea (*Pisum sativum* L.) cv. ‘Frisson’ on the matrix composition of infection droplets in nodule cells; Table S1: List of differentially expressed genes in 20-day-old nodules of pea (*Pisum sativum* L.) plants cv. ‘Frisson’ treated with Imidor Pro insecticide in the concentration recommended by the manufacturer.
