# Supplementary material for: Effect of Insecticides Imidacloprid and Alpha-Cypermethrin on the Development of Pea (Pisum sativum L.) Nodules
Source: Plants (Basel). 2024 Dec 7;13(23):3439. doi: 10.3390/plants13233439 (PMC11644560; doi:10.3390/plants13233439)
Supplement: Supplementary file 1 [file plants-13-03439-s001.zip › plants-3335741-supplementary.pdf]

Electronic Supplementary Material

Effect of Insecticides Imidacloprid and Alpha-Cypermethrin on the Development of Pea (*Pisum sativum* L.) Nodules

Artemii P. Gorshkov[1,&], Pyotr G. Kusakin[1,&], Maxim G. Vorobiev [2], Anna V. Tsyganova[1,*] and Viktor E. Tsyganov[1]

[1] All-Russia Research Institute for Agricultural Microbiology, Laboratory of Molecular and Cell Biology, Saint Petersburg, Russia; a.gorshkov@arriam.ru (A.P.G.); pyotr.kusakin@arriam.ru (P.G.K.); vetsyganov@arriam.ru (V.E.T)

[2] Research Resource Centre "Molecular and Cell Technologies", Saint Petersburg State University, 199034 Saint Petersburg, Russia; vorobiev.maxim@spbu.ru (M.G.V.)

[&] These authors contributed equally to this work

*Author for correspondence:

Anna V. Tsyganova

Tel: +7 812 4705100

E-mail: avtsyganova@arriam.ru

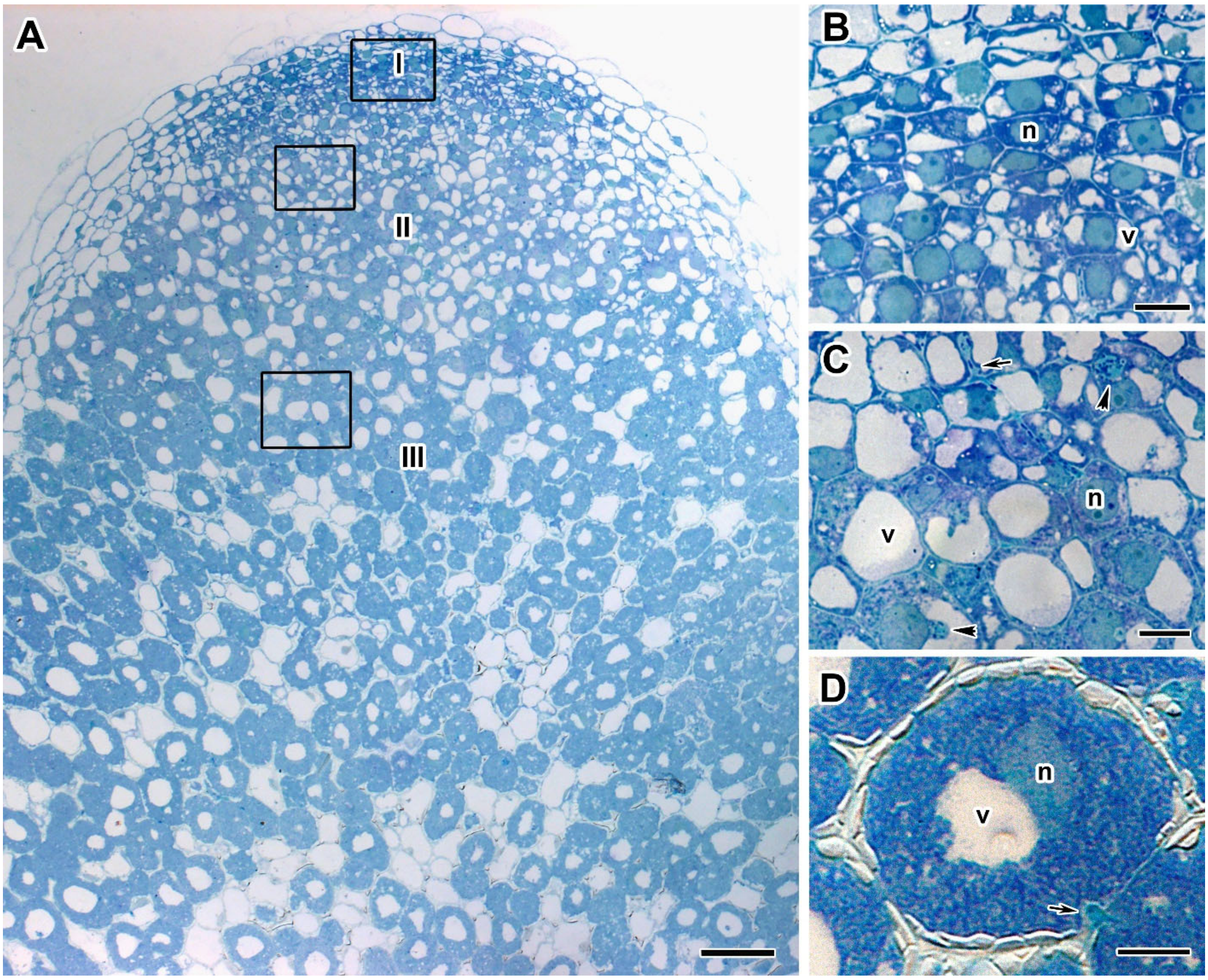

Supplementary Figure 1. Histological organization of the nodules of 20-day-old untreated plants of pea (*Pisum sativum* L.) cv. 'Frisson'. (**A**) Longitudinal section of a nodule. (**B**–**D**) High magnification of the boxed area in (**A**). (**B**) Nodule meristematic cells. (**C**) Cells in the infection zone. (**D**) Infected cells in the nitrogen fixation zone. I, meristem; II, infection zone; III, nitrogen fixation zone; n, nucleus; v, vacuole. Arrows indicate infection threads, arrowheads indicate infection droplets. Bars (**A**) = 100 μm, (**B**–**D**) = 10 μm.

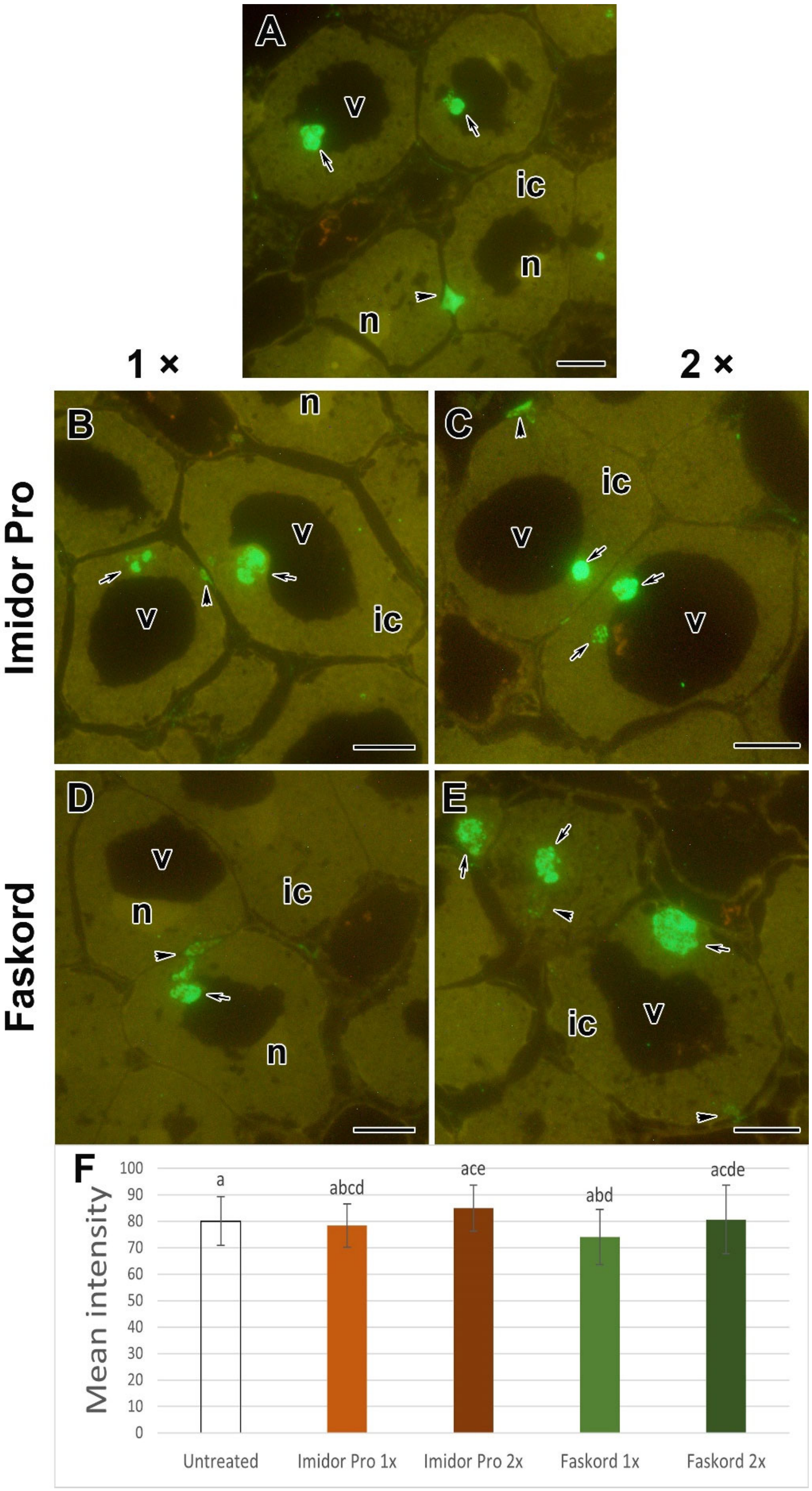

Supplementary Figure 2. Effect of insecticide treatment of pea (*Pisum sativum* L.) cv. 'Frisson' on the matrix composition of infection droplets in nodule cells. (**A**–**E**) Immunolocalization of intercellular plant glycoprotein labeled with MAC265 MAb. (**A**) Section of nodules of untreated plant. Treatment with Imidor Pro (**B**,**C**) and Faskord (**D**,**E**) with concentration recommended by the manufacturer (**B**,**D**) and with a twofold-concentrated solution of insecticides (**C**,**E**). (**F**) Mean fluorescence intensity. The secondary antibodies used were goat anti-rat (**A**–**E**) IgG MAb conjugated with Alexa Fluor 488. ic, infected cell; n, nucleus; v, vacuole. Arrows indicate infection droplets. Different letters indicate groups with a significant difference according to Tukey's HSD test (p-value < 0.05; n = 30–45). Vertical bars represent standard deviation. Bars = 10 μm.

Supplementary Table 1. List of differentially expressed genes in 20-day-old nodules of pea (*Pisum sativum* L.) plants cv. 'Frisson' treated with Imidor Pro insecticide in the concentration recommended by the manufacturer.

Gene ID	log2 fold change	Annotation
Psat2g009480	6.517476746	Pentatricopeptide repeat
Psat3g090720	3.66947919	Phosphatidylethanolamine-binding protein
Psat1g014320	3.334807949	Transglycosylase SLT domain
Psat7g168680	3.090087872	Leucine rich repeat
Psat5g092480	2.953520429	Major Facilitator Superfamily
Psat2g101000	2.889256342	Heavy metal transport/detoxification protein
Psat5g016280	2.624866624	Cytochrome P450
Psat2g026280	2.563901004	Xylanase inhibitor N-terminal
Psat1g194520	2.419249572	Fasciclin domain
Psat6g013480	2.358409332	EF-hand calcium-binding domain profile.
Psat1g068040	2.259714437	UDP-glucoronosyl and UDP-glucosyl transferase
Psat2g133560	2.245046656	SAM dependent carboxyl methyltransferase
Psat7g122840	2.218827316	Plastocyanin-like domain
Psat6g069040	2.131841851	C2H2-type zinc finger
Psat7g247800	2.089274401	Multicopper oxidase
Psat3g204320	2.08814167	COBRA-like protein
Psat7g068160	2.073451602	Fasciclin domain
Psat7g224400	2.053595092	Fasciclin domain
Psat4g015520	2.040841564	Cytokinin dehydrogenase 1 + FAD and cytokinin binding
Psat0s2363g0160	1.982008256	Mechanosensitive ion channel
Psat2g144000	1.974032731	Transferase family
Psat1g050520	1.957079734	Cation transport protein
Psat1g195520	1.939432991	EamA-like transporter family
Psat7g068120	1.894416828	Intrinsic to membrane
Psat0s1086g0040	1.865485021	Protein of unknown function + DUF547
Psat6g021360	1.833076065	Cytochrome P450
Psat0s3979g0120	1.811252565	Late embryogenesis abundant protein
Psat3g124040	1.793509408	Dirigent-like protein
Psat5g277720	1.770657325	Fasciclin domain
Psat2g114040	1.766342847	Cupin
Psat1g128520	1.759610412	COBRA-like protein
Psat0s2012g0040	1.754841636	DOMON domain
Psat2g074320	1.674060319	Cellulase (glycosyl hydrolase family 5)
Psat6g061600	1.628012371	Cellulose synthase
Psat4g125440	1.589225235	Cellulose synthase
Psat5g299160	1.560435457	Sulfate permease family
Psat1g047000	1.550779222	ABC transporter transmembrane region
Psat5g019840	1.537132361	Transmembrane amino acid transporter protein
Psat0s2127g0040	1.507836669	Unknown gene
Psat5g249800	1.499711977	Glycosyl transferase family 8
Psat2g127960	1.474155676	Cellular response to abscisic acid stimulus
Psat1g149960	1.45802971	Cellulose synthase
Psat7g006400	1.431647172	Cellulose synthase

Psat6g230840	1.418182589	Family of unknown function (DUF716)
Psat5g003080	1.415381299	Cyclophilin type peptidyl-prolyl cis-trans isomerase/CLD
Psat1g174280	1.388974569	Microtubule-associated protein 70
Psat1g121240	1.382296161	Cotton fibre expressed protein
Psat2g141040	1.368659598	UDP-glucoronosyl and UDP-glucosyl transferase
Psat7g091280	1.345144343	Unknown gene
Psat4g037680	1.340541293	Nucleoside transporter
Psat1g041680	1.316005999	Unknown gene
Psat1g152520	1.313398593	Non-haem dioxygenase in morphine synthesis N-terminal
Psat4g024560	1.29517741	ansferase activity + transferring phosphorus-containing groups
Psat1g216120	1.275797909	Caspase domain
Psat1g159520	1.243043113	Unknown gene
Psat7g123800	1.217410813	Phloem protein 2
Psat2g154000	1.213997566	Thaumatin family
Psat5g277760	1.194967799	Fasciclin domain
Psat7g027320	1.173888394	Unknown gene
Psat7g068960	1.171816721	Cellulose synthase
Psat5g091000	1.171321811	Unknown gene
Psat7g137440	1.118773636	Phloem protein 2
Psat6g192400	1.11498624	ABA/WDS induced protein
Psat2g023080	1.104200897	Unknown gene
Psat4g024360	1.101616062	Unknown gene
Psat6g021600	1.094327524	Unknown gene
Psat2g057640	1.077318038	Jacalin-like lectin domain
Psat7g027360	1.076894579	Unknown gene
Psat1g160280	1.071976539	Unknown gene
Psat5g091040	1.0678506	Unknown gene
Psat1g126560	1.061328046	MADS domain signature
Psat5g085520	1.042862486	Transmembrane amino acid transporter protein
Psat5g250680	1.040708016	Protein of unknown function (DUF1685)
Psat1g216240	1.039070709	Caspase domain
Psat5g020720	1.03212403	Transmembrane amino acid transporter protein
Psat6g012520	0.996879535	Response to temperature stimulus
Psat0s302g0200	0.992840824	Myb-like DNA-binding domain
Psat4g024520	0.968617383	ansferase activity + transferring phosphorus-containing groups
Psat2g131520	0.966828825	Domain of unknown function (DUF2828)
'sat0s5147g0040	0.966565539	N-terminal C2 in EEIG1 and EHBP1 proteins
Psat5g091920	0.965459754	Unknown gene
Psat5g157360	0.955000133	Protein tyrosine kinase
Psat7g163680	0.910260452	Lipase (class 3)
Psat7g130840	0.878564446	Phloem protein 2
Psat7g130600	0.873355579	Phloem protein 2
Psat2g131480	0.767945636	Domain of unknown function (DUF2828)
Psat5g105200	0.761626894	O-methyltransferase
Psat1g004760	0.759476341	Polysaccharide biosynthesis
Psat7g014920	0.748149502	Myc-type + basic helix-loop-helix (bHLH) domain profile.

Psat3g033760	0.702492709	Phosphatidylethanolamine-binding protein
Psat7g097360	0.693610655	EamA-like transporter family
Psat2g142680	0.687569726	Alternative oxidase
Psat7g204640	0.66383094	Fantastic Four meristem regulator
Psat1g041960	0.613166982	Myc-type + basic helix-loop-helix (bHLH) domain profile.
Psat5g125160	0.601938416	Protein of unknown function (DUF3633)
Psat7g145280	0.553598336	Protein kinase domain
Psat4g225160	0.527915128	Membrane transport protein
Psat1g078480	0.448654267	Cytochrome P450
Psat7g048520	−3.628169905	Ring finger domain
Psat4g225000	−2.946880632	AP2 domain
Psat1g194920	−2.293939688	Tify domain
Psat0s4837g0040	−2.264948322	Unknown gene
Psat7g212000	−2.081537343	Unknown gene
Psat3g023040	−1.469289168	Aromatic amino acid lyase
Psat3g056880	−2.034896427	Myb-like DNA-binding domain
Psat7g114080	−1.994999499	PDDEXK-like family of unknown function
Psat3g011600	−1.943893436	WIF domain profile.
Psat3g023120	−1.482479145	Aromatic amino acid lyase
Psat3g162200	−1.878168088	Unknown gene
Psat5g118840	−1.87438766	Unknown gene
Psat5g119040	−1.853952839	Unknown gene
Psat5g232880	−1.806792229	Unknown gene
Psat1g046920	−1.574664053	Aromatic amino acid lyase
Psat0s9163g0040	−1.796070476	Unknown gene
Psat5g022440	−1.771675772	Transferase family
Psat0s1627g0040	−1.707646227	Myb-like DNA-binding domain
Psat3g180120	−1.67800411	Unknown gene
Psat5g089720	−1.677929215	C2 domain
Psat0s3829g0080	−1.594811723	Domain of unknown function (DUF4228)
Psat6g055240	−1.797611016	AP2 domain
Psat1g131280	−1.889932491	Glycosyl hydrolases family 18
Psat5g133080	−2.075946023	EamA-like transporter family
Psat4g086600	−1.416575573	Protein kinase domain
Psat4g094680	−1.320480667	Plasma membrane
Psat5g089800	−1.313102274	C2 domain
Psat0s1849g0080	−1.308204495	CCT motif
Psat0s766g0040	−1.228532156	Tify domain
Psat7g227560	−1.04778838	Sugar (and other) transporter
Psat5g200320	−0.929383176	Response regulator receiver domain
Psat6g081800	−0.890075133	GDSL-like Lipase/Acylhydrolase
Psat6g120040	−0.612628167	CobN/Magnesium Chelatase
Psat1g122080	−0.560281141	Trypsin and protease inhibitor
Psat3g194360	−0.450432998	Transmembrane amino acid transporter protein
